# Root Projection of One-Sided Time Series

**DOI:** 10.6028/jres.096.018

**Published:** 1991

**Authors:** John A. Simmons

**Affiliations:** National Institute of Standards and Technology, Gaithersburg, MD 20899

**Keywords:** causal boundary roots, Gram-Schmidt algorithm, Prony’s method, root projection, signal processing, time series, Z transforms

## Abstract

Until recently it has been impossible to accurately determine the roots of polynomials of high degree, even for polynomials derived from the Z transform of time series where the dynamic range of the coefficients is generally less than 100 dB. In a companion paper, two new programs for solving such polynomials were discussed and applied to signature analysis of one-sided time series [1], We present here another technique, that of root projection (RP), together with a Gram-Schmidt method for implementing it on vectors of large dimension. This technique utilizes the roots of the Z transform of a one-sided time series to construct a weighted least squares modification of the time series whose Z transform has an appropriately modified root distribution. Such a modification can be employed in a manner which is very useful for filtering and deconvolution applications [2]. Examples given here include the use of boundary root projection for front end noise reduction and a generalization of Prony’s method.

## 1. Introduction

Let *y* be a complex number. We define $\hat{y}$ to be the “geometric sequence” vector with components $\hat{y_{n}}=y^{n},\;n=0,\ldots ,N$. Then given a time series of length *N*+1 represented by the vector $a:N,\;a\left(y\right)={\hat{y}}^{T}a$; and the condition that *y* be a root of *a*(*y*) is:

$${\hat{y}}^{T}a=0.$$ (1)

Given a (possibly complex) time series ***a:N*** and any set of complex roots of eq (1)*y*_1_,…, *y_M_*, *M*≤*N*, we shall construct a linear projection to modify ***a:N*** to another time series ***b:N*** so that *b*(*y*_α_) = 0, *α* = 1,…, *M*, and so that

$${\left(a-b\right)}^{T}G\left(a-b\right)*,$$ (2)

where “*” means complex conjugate, is a minimum for any positive definite Hermitian weighting matrix *G.* In other words ***b:N*** is the series closest to ***a:N*** in a weighted least squares sense, which has a chosen set of complex numbers among the roots of its *Y* transform. We refer to this process as *root projection* (RP).

In a slightly more general context consider a (possibly complex) time series ***a:N***, a set of complex numbers *y*_1_…, *y_m_* with *M*≤*N*, and a set of complex values υ_1_,*…*, *υ_m_.* We construct a time series ***b:N*** whose difference from ***a:N*** is minimal among those series with *b* (*y_α_*) =*υ*_*α*_, *α* = 1,…, *M*.

Because of the slight complication due to the complex numbers involved, we shall set and solve the above minimization problem using Lagrangian multipliers. We want to find the time series ***b:N*** such that:

$$\begin{array}{l} b:N{=}_{x:N}^{\min}[{\left(a-x\right)}^{T}G^{T}\left(a-x\right)*+\\ \sum_{\alpha =1}^{M}\lambda_{\alpha }^{*}\left(x^{T}\hat{y_{\alpha }}-\upsilon_{\alpha }\right)+\sum_{\alpha =1}^{M}\lambda_{\alpha }\left(x{*}^{\hat{T}}y_{\alpha }^{*}-\upsilon_{\alpha }^{*}\right)]. \end{array}$$ (3)

Using index notation and differentiating eq (3) with respect x_j_* and $\lambda_{\alpha }^{*}$, we have:

$$\begin{array}{l} G_{ki}\left(a_{i}-b_{i}\right)=\lambda_{\beta }{\left(y_{\beta }^{*}\right)}^{k}\\ b_{k}{\left(y_{\alpha }\right)}^{k}=\upsilon_{\alpha }. \end{array}$$ (4)

Setting $y_{\alpha k}\equiv {\left(y_{\alpha }\right)}^{k},\;y_{\alpha k}^{*}\equiv {\left(y_{\alpha }^{*}\right)}^{k},$ and $G_{ij}^{-1}\equiv {\left(G^{-1}\right)}_{ij};$ solving for *b_i_* in the first of eqs (4), substituting into the second equation and solving for *λ_ß_*:

$$\begin{array}{l} \lambda_{\beta }=Q_{\beta \alpha }^{-1}\left(a_{j}y_{\alpha j}-\upsilon_{\alpha }\right),\;\text{where}\\ Q_{\alpha \beta }=y_{\alpha k}G_{kl}^{-1}y_{\beta l}^{*} \end{array}$$ (5)

whence:

$$b_{i}=a_{i}-a_{j}y_{\alpha j}Q_{\beta \alpha }^{-1}y_{\beta m}^{*}G_{mi}^{-1}+\upsilon_{\alpha }Q_{\beta \alpha }^{-1}y_{\beta m}^{*}G_{mi}^{-1}.$$ (6)

To make the connection to linear vector space theory, we start with complex *N* space, ${ℭ}^{N}$, having an inner product given by the positive-definite Hermitian matrix *G:*

$$\left(a,b\right)\equiv a^{T}G^{T}b^{*},$$ (7)

and complex *M* space, ${ℭ}^{M}$, having an inner product given by the Hermitian matrix *Q*^−1^ (assumed, here, to be non-singular) with an inner product definition similar to eq (7) and with vectors such as ***υ*** (with components *υ_α_, α* = 1,…, *M*) [3]. We define the mapping *Y* from ${ℭ}^{N}$ to ${ℭ}^{M}$ by:

$$Y\equiv \left[Y_{\alpha i}=y_{\alpha i};\;\alpha =1,\ldots ,M,\;i=1,\ldots ,N\right].$$ (8)

*Y* has an adjoint mapping *Y*^†^, from ${ℭ}^{M}$ to ${ℭ}^{N}$ given by the condition ${\left(Y^{†}\upsilon \right)}^{T}G^{T}a^{*}=\upsilon^{T}{\left(Q^{-1}\right)}^{T}{\left(Ya\right)}^{*}$, from which

$$Y^{†}=G^{-1}Y^{*T}Q^{-1}.$$ (9)

In matrix notation eq (6) becomes:

$$\begin{array}{l} b=\left(I-P\right)a+Y^{†}\upsilon \\ P=Y^{†}Y,P^{2}=P, \end{array}$$ (10)

the projection property, *P*^2^*=P*, following directly from eq (6). It also follows directly from eq (6) that the range of *I*−*P* is contained in the null space of *Y*^†^, while the range of *P* is contained in the range of *Y*^†^. Since both *I−P* and *P* as well as the null space of *Y* and the range of *Y*^†^ decompose ${ℭ}^{N}$ (the latter being an orthogonal decomposition), *I−P* and *P* are orthogonal mappings onto the respective spaces. The mapping *I−P* is, then, the desired root projection.^1^

## 2. A Gram-Schmidt Algorithm for Root Projection

In order to carry out root projection, we need an orthogonal basis for the range of *Y*^†^ in the unitary space with metric *G*. From eq (8) we see that this space is spanned by vectors of the form $G^{-1}\hat{y_{\alpha }}$. For any two such vectors:

$${\left(G^{-1}\hat{y_{\alpha }}\right)}^{T}G^{T}\left(G^{-1}\hat{y_{\beta }}\right)*={\left(G^{-\frac{1}{2}}\hat{y_{\alpha }}\right)}^{T}\left(G^{-\frac{1}{2}}\hat{y_{\beta }}\right)*,$$ (11)

where

$${\left(G^{-\frac{1}{2}}\right)}^{2}=G^{-1}.$$ (12)

Thus, an orthonormal basis chosen from the vectors $G^{-\frac{1}{2}}\hat{y_{\alpha }}$ by applying the Gram-Schmidt process using the ordinary unitary inner product will determine an orthogonal basis for the range of *Y*^†^ in the unitary space with metric *G* by multiplying each vector by *G*^−½^. However, when only a limited number of vectors need to be projected, the projection can be carried out more efficiently by:

$$\begin{array}{l} i)\;\text{transforming the vector}\;a\;\text{to}\;G^{\frac{1}{2}}a\\ ii)\;\text{projecting}\;G^{\frac{1}{2}}a\;\text{in the Euclidean norm using the modified basis}\;G^{\frac{1}{2}}\hat{y_{\alpha }}\\ iii)\;\text{transforming the result back by multiplication with}\;G^{-\frac{1}{2}}. \end{array}$$ (13)

The root projection process is especially simple when the set of *y_α_*’s is closed under complex conjugation (i.e., if *y_α_* belongs to the set, so does $y_{\alpha }^{*}$) for the case of simple time or frequency weighting. Because of the closure under complex conjugation, the $\hat{y_{\alpha }}$ vectors can be replaced by the vectors consisting of their real and imaginary parts, $ℜ\left(\hat{y_{\alpha }}\right)$ and $J\left(\hat{y_{\alpha }}\right)$. By the time weighting case we mean that *G*^−1^ and *G*^−½^ are diagonal (and, therefore, positive and real), so that no unitary transformation is required. In that case each of the rows is multiplied by the same factor. Thus, the basis for the range of *Y*^†^ can be obtained by applying the real form of the Gram-Schmidt process to the vectors $ℜ\left(G^{-\frac{1}{2}}\hat{y_{\alpha }}\right)$ and $J\left(G^{-\frac{1}{2}}\hat{y_{\alpha }}\right)$. The root projection of a real time series, outlined in eq (13), will, in this case, also be real.

In the case of frequency weighting we mean scalar weights applied to the DFT components of a time series. The DFT process is, itself, a unitary transformation, and the orthogonalization process can be made real by the trick of putting $ℜ\left({\bar{a}}_{k}\right)\sqrt{2}$ in place of the DFT component ${\bar{a}}_{k}$ and putting $J\left({\bar{a}}_{k}\right)\sqrt{2}$ in place of the DFT component $\tilde{a_{-k}}$. The real dot product of these series is the same as the unitary dot product of the DFT’s. A diagonalized frequency weighting metric can then be applied to these transformed DFT series and the diagonalization process carried out. If projection is to be carried out on a real time series, it can be done in these transformed coordinates, otherwise, the new orthogonal DFT coordinates have to be reconstructed before doing the projection. Of course, the projected DFT series has to be reconverted to time series form after projection.

The real form of root projection can also be obtained by using QR algorithms such as given in LINPACK [4]. However, the implementation of the QR algorithms in LINPACK is CPU core consuming for large *N* and does not easily allow storage of the orthogonal matrix. These disadvantages are overcome using the Gram-Schmidt method given above. Employing a form of the Gram-Schmidt algorithm suggested by G.W. Stewart [5,6], a dynamically dimensioned Fortran 77 code was constructed allowing efficient use of a user selected buffer with the option of saving the orthogonalized basis vectors on disc for rapid repeated projection [7]. For *y_α_* with modulus greater than one, projection was carried out using the root reciprocal to avoid overflow. In this case the series starts at *y_α_^−N^* and goes up to 1, a procedure equivalent to reversing the series to be projected.

## 3. Examples

We have principally used root projection for deconvolution. That topic is discussed in the third paper in this series [2]. Here we give four illustrative examples of root projection. The fourth example, applying root projection to Prony’s method may have some practical use. However, a detailed study has not been conducted.

**Figure 1:**
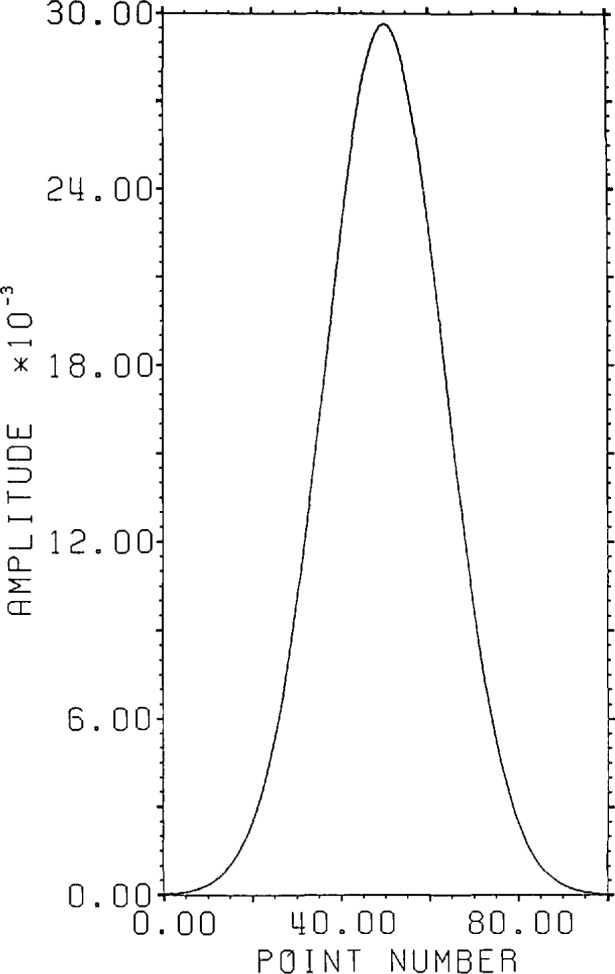
Normalized 101 point Gaussian with 60 dB dynamic range.

**Figure 2:**
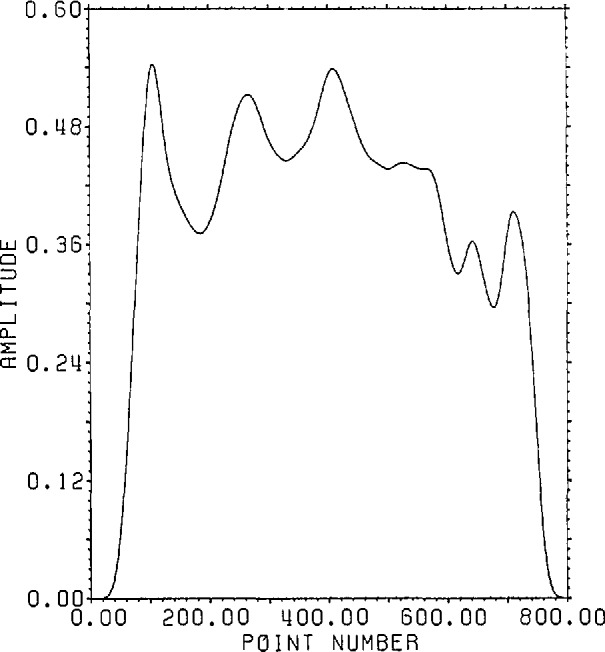
Convolution of the first 700 points of the time series in figure 2.4a of reference [1] with the 101 point time series of figure 1.

**Figure 3:**
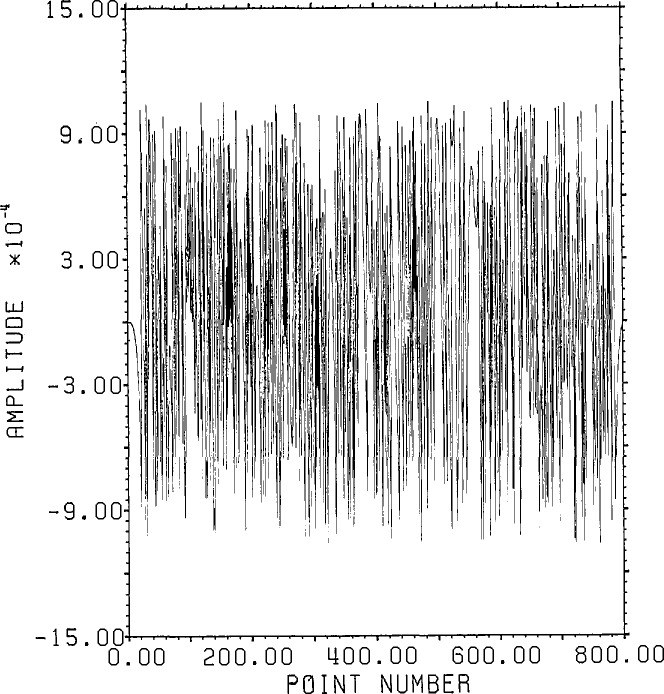
8-bit roundoff noise for the series in figure 2.

**Figure 4:**
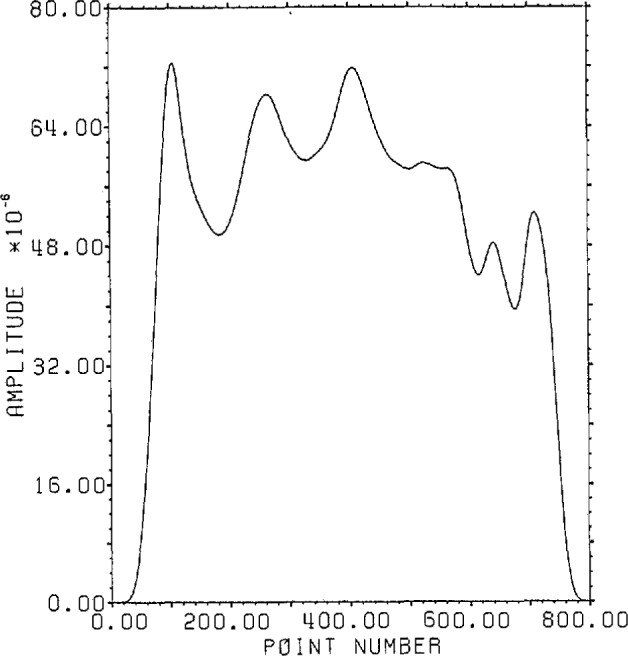
Error from projecting the series in figure 2 plus noise through all roots of the *Y* transform of the series in figure 2. Note that the remaining error is proportional to the original series.

**Figure 6:**
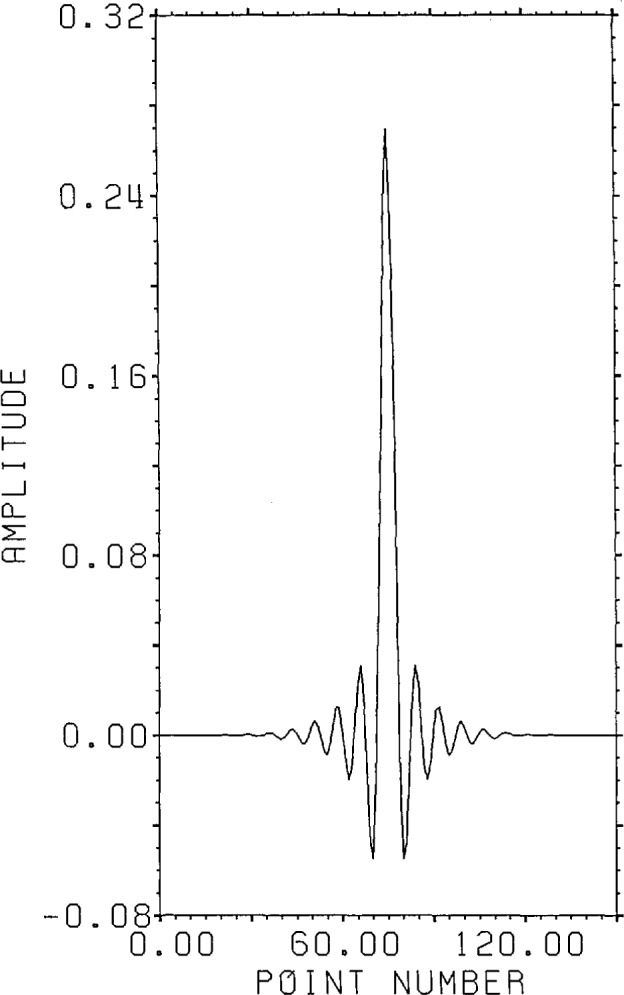
Optimal maximal ripple filter of 151 elements.

**Figure 7:**
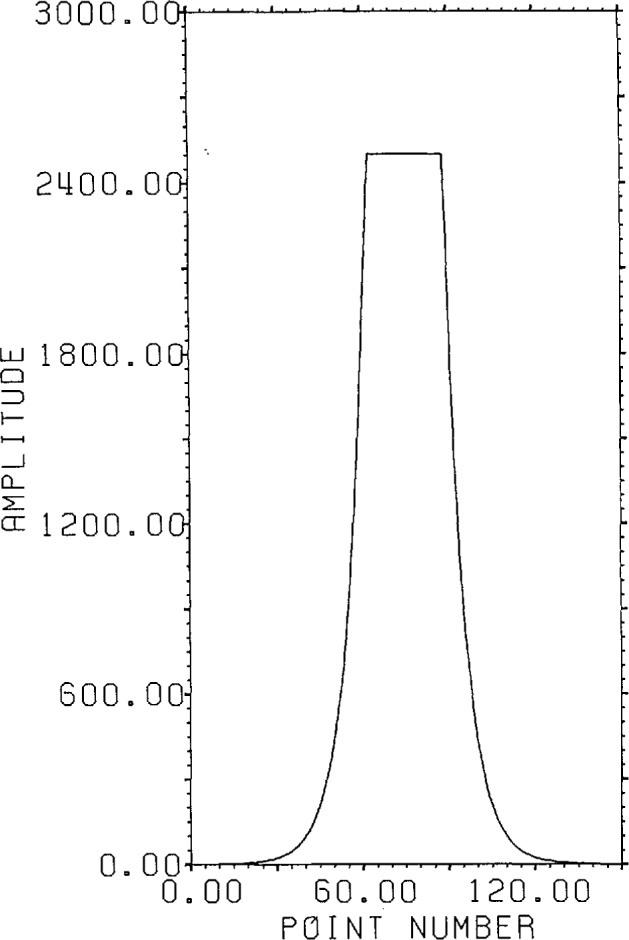
Time weights for approximating an optimal filter.

**Figure 8:**
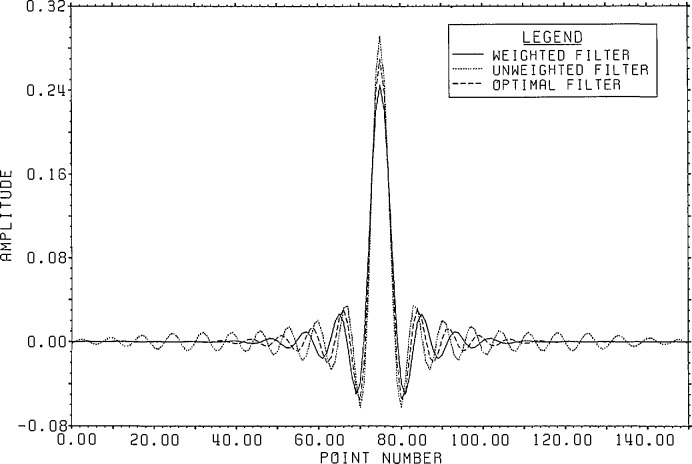
Comparative time plot showing the effects of using weighted projection for approximating an optimal filter.

**Figure 9:**
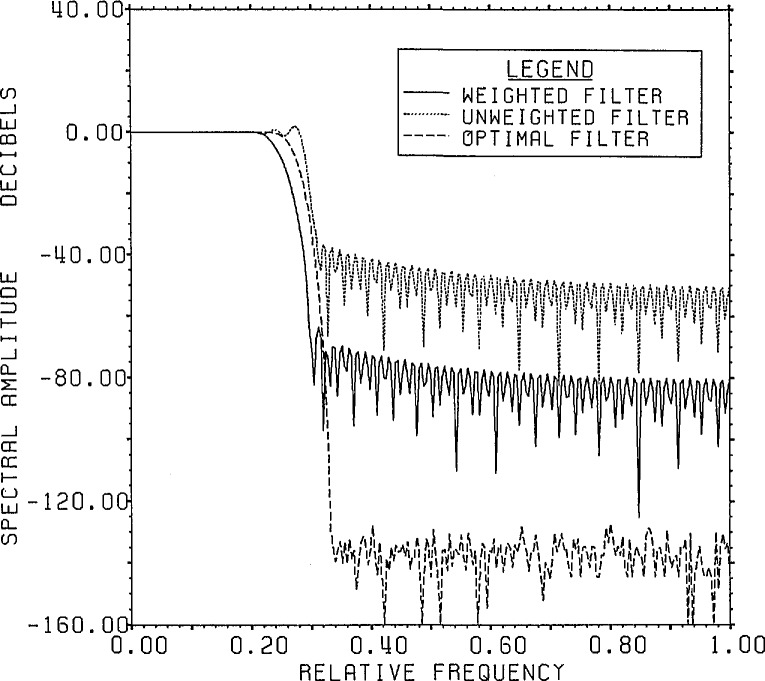
Comparative frequency plot showing the effects of using weighted projection for approximating an optimal filter.

**Figure 10:**
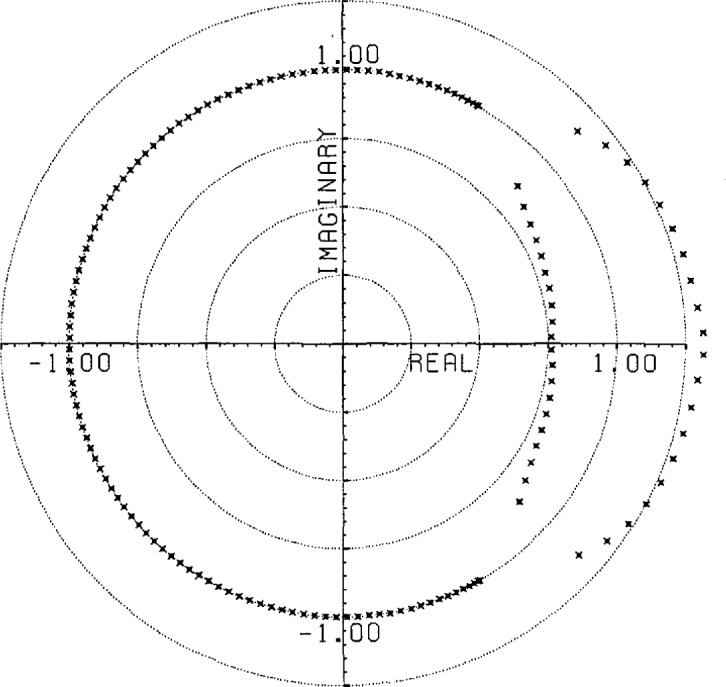
Roots of the *Y* transform of the optimal filter shown in figure 6 showing roots in the stopband.

**Figure 11:**
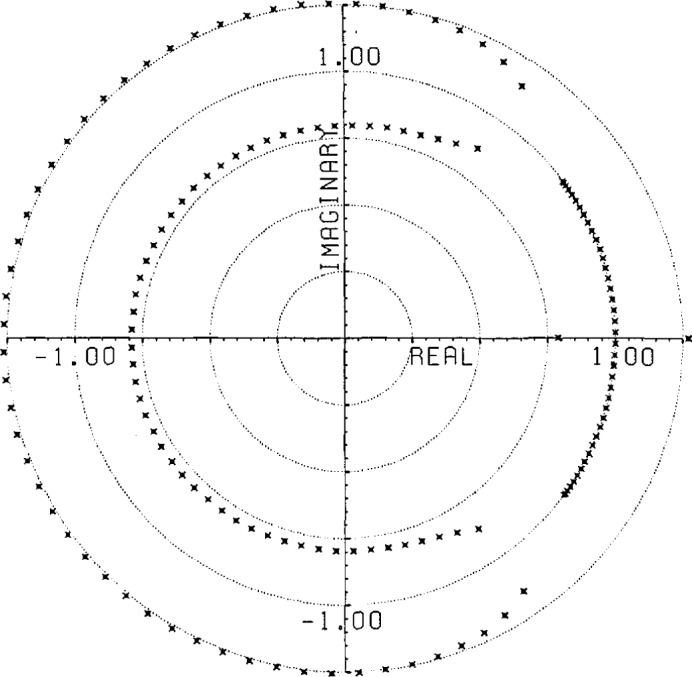
Roots of the *Y* transform of the optimal filter of figure 6 minus the shifted delta series showing roots in the pass band.

1. Figure 1 shows a normalized 101 point Gaussian with 60 dB dynamic range (ratio of center to edge points). In figure 2 we show the 800 point result of convolving the first 700 points of the experimental waveform of figure 2.4a in [1] with the Gaussian of figure 1. The noise pattern shown in figure 3 results from rounding off the time series in figure 2 to 8 bit accuracy. The rounded-off time series is not shown. The standard deviation of this noise is 6.02×10^−4^.
  A basis for all 800 point time series can be formed from the waveform of figure 2 together with the 799 geometric root vectors formed from the roots of the *Y* transform of that waveform. The noise vector of figure 3 must then be a linear combination of all these vectors, while the true signal vector of figure 2, which is one of the basis elements, is orthogonal to all of the 799 geometric root vectors. If we use the geometric root vectors built from the Gaussian of figure 1 and apply root projection to the rounded-off series built from the series in figure 3, the noise of the resultant series will be reduced in magnitude, since it is orthogonal to the 100 geometric root vectors formed from the roots of the *Y* transform of the Gaussian. The standard deviation of that noise is 5.6×10^−4^, almost exactly the theoretical estimate of ${\left(\frac{7}{8}\right)}^{\frac{1}{2}}$ expected for Gaussian noise. If the 799 geometric root vectors from both the Y transforms of the Gaussian and the waveform of figure 2.4a in [1] are used for the projection, then the error appears as in figure 4—a mini-image of the correct time series—with a standard deviation of 2.0×10^−5^ (again approximately ${\left(800\right)}^{-\frac{1}{2}}$ of the original noise deviation). This time, of course, the noise is biased with a mean value of 4.9 × 10^−5^. This curve also indicates the precision of the root finding and Gram-Schmidt routines used in these calculations.
2. The use of an extensive causal boundary root set for front end filtering is shown in figures 5a and 5b. To the Gaussian filtered curve of figure 2.3 a in [1] we add the −40 dB noise distribution shown in figure 5a and apply root filtering using only the causal boundary roots from figure 2.1b of [1]. The noise after filtering showing extensive front end reduction is shown in figure 5b.
3. A more general example of root projection employing both time and frequency weighting is afforded by constructing an approximation to an optimal maximal-ripple lowpass filter. Figure 6 shows such a filter of 151 elements designed using the Remez exchange algorithm of McClellan et al. [8]. In this case the passband was set for 20% of the Nyquist frequency and the stopband for frequencies over 33% of the Nyquist frequency.
  We start with a delta series shifted so that the value 1.0 lies at position #76 in the Center of the time interval. To use unweighted projection and 101 roots evenly spaced from −60° to +60° on the unit circle would produce the familiar series obtained from windowing the DFT of the shifted delta series. However, by using time weighted projection with the center symmetric time weight shown in figure 7 and adding two extra roots near each edge of the stopband region, one can produce a filter with 120 dB attenuation in the stopband and ~2.5×10^−7^ leakage in the time domain, but with 0.11 ripple in the passband. Without time weighting the filter produced has 64 dB attenuation in the stop band and 0.08 ripple in the passband, but with ~1.0×10^−3^ leakage in the time domain.
  To remove the passband ripple from the weighted filter we subtract from it the shifted delta series. The resulting series should be close to zero in the passband region. We place 31 roots evenly in the passband and again place two extra pairs of roots near the edge of the band. Applying frequency weighted projection with a simple square frequency weight to hold the stopband attenuation in place, we obtain (after re-adding in the shifted delta series) the doubly root projected filter shown in figure 8, where it is compared with the unweighted doubly projected and optimal filters. The weighted series closely resembles the optimal series of figure 6 with leakage at the ends of the time interval of ~8.0×l0^−5^ as opposed to ~2.0×10^−3^ for the unweighted filter. The leakage for the optimal filter is ~6.0×10^−8^. The attenuation in the stopband is 70 dB and the ripple in the passband is 1.0×10^−4^ for the weighted filter while the unweighted filter has 36 dB attenuation and 8.0×10^−4^ ripple. The optimal filter has 116 dB attenuation and 5.0×10^−5^ ripple. The spectra for the three filters are compared in figure 9.
  The root patterns in the stopband for the optimal filter are shown in figure 10. As can be seen, they also cluster near the edge of the stopband, but they are not evenly spaced throughout the stopband. Similarly, the roots of the optimal filter minus the shifted delta series, shown in figure 11, exhibit the same uneven spacing and clustering in the passband region.
4. Root projection can be used to provide a “global” least squares generalization to the Prony method. Prony’s method is applied to the case of *N+*1 data points which one wants to represent as the sum of *n* decaying exponentials (Prony takes *N*+1 even and *n* = (*N+*1)/2):
  $$F_{k}=\sum_{j=1}^{n}A_{j}y_{j}^{k},\;k=0,\ldots ,N.$$ (14)
  Here *y_j_* is thought or as a complex number of modulus less than 1, $\;y_{j}=e^{\beta_{j}T}$, real(*β_j_*)<0, and *T* a sampling interval (See, e.g., [9]). Eq (14) can be rewritten, taking advantage of the cyclotomic polynomial expression, as
  $$F\left(y\right)=\sum_{k=0}^{N}F_{k}y^{k}=\sum_{j=1}^{n}A_{j}\frac{1-{\left(y_{j}y\right)}^{N+1}}{1-y_{j}y}.$$ (15)

Combining the terms in eq (15) together to form a single fraction gives

$$\begin{array}{l} F\left(y\right)=[A_{1}\left(1-y_{2}y\right)\cdots \left(1-y_{n}y\right)\left(1-{\left(y_{1}y\right)}^{N+1}\right)+\ldots \\ \;+A_{n}\left(1-y_{1}y\right)\cdots \left(1-y_{n-1}y\right)\left(1-{\left(y_{n}y\right)}^{n+1}\right)]/\\ \;\left[\left(1-y_{1}y\right)\cdots \left(1-y_{n}y\right)\right]. \end{array}$$ (16)

Equation 16, then, has the form

$$F\left(y\right)=\frac{P\left(y\right)+y^{N+1}R\left(y\right)}{Q\left(y\right)},$$ (17)

where *Q*(*y*) is a polynomial of degree *n* whose roots are (*y_j_*)^−1^, and *P*(*y*)*+y^N+^*^1^*R*(*y*) is a polynomial of degree *N+n* whose *N+*1*−n* coefficients from that of *y^n^* to that of *y^N^* are zero. The roots of *Q*(*y*) are referred to as the poles of *F.* Conversely one can show that if eq (17) holds with the corresponding coefficients zero, then setting

$$\begin{array}{l} Q\left(y\right)=\prod_{j=1}^{n}\left(1-y_{j}y\right)\\ A_{j}=-\frac{P\left(1/y_{j}\right)}{Q\prime \left(1/y_{j}\right)} \end{array}$$ (18)

yields the expression 14.

The goal, then, is to solve the algebraic equation:

$$Q\left(y\right)F\left(y\right)=P\left(y\right)+y^{N+1}R\left(y\right),$$ (19)

where *P, Q*, and *R* are all unknown. Only the integer *n* is given. This is generally a very ill-conditioned process, but when the *y_j_*’s are real or occur in conjugate pairs, the following root projection procedure is often successful:

- Use an initial estimate for the coefficients, *c_i_*, of *P+y^N+^*^1^*R* by setting *c_i_ = κ*_1_ for *i* =0,…, *n*−1, *c_i_* =0 for *i=n,…, N*, and *c = κ*_2_ for *i=N+*1,*…, N+n*; and use for the diagonal weighting matrix, *W*, the series *w_l_* with *w_l_ = λ*_1_ for *l* = 1,*…, n, w_l_* = 1 for *l=n+*1,*…, N+*1, and *w*_1_ *= λ*_2_ for *l =N +*1,…, *N+n+*1, where *κ*_1_, *κ*_2_, *λ*_1_, and *λ*_2_ are user selected values.
- Project the series made from *P+y^N+^*^1^*R* into the range of *F* using *W* and the geometric row vectors made from the roots of *F*(*y*). The projected polynomial will have negligible values for the coefficients between n+1 and *N*+1 (depending on the noise in the data) and will be divisible by *F*(*y*). Further, *P*(*y*) can be read off from the projection and *Q*(*y*) found by simple division (using FFT methods, for instance, as discussed in [2]).

As long as: *i*) the projection is stable, *ii*) the projected vector is not zero, and *iii*) the values of the weights are large enough to produce an answer within the noise for similar cases with no data noise, then the values of *κ*_1_, *κ*_2_, *λ*_1_, and *λ*_2_ have very little effect on the calculated *y_j_*’s and *A_j_*’s. In the ordinary Prony case there are as many degrees of freedom added in *R* as are restricted between *P* and *R* (one degree is an irrelevant multiplicative constant since *F* is expressed as a ratio).

This method has been tried for several examples with both real and complex poles with maximum to minimum root amplitude ratios up to 60:1 and with minimum root separation down to 0.01. Using double precision data (with *κ*_1_ = 10^8^, *κ*_2_=10^4^, *λ*_1_=10^28^, and *λ*_2_ = 10^18^) it works well up to about eight poles after which the positions of the larger poles start to degenerate (due to the ill-conditioning of the problem). The representation of the time series coefficients of *F* remains accurate to a relative error of about 10^−12^, which represents approximately the cumulative accuracy of the algorithms involved. Using data given to 16 bit precision (around 5 place accuracy), the method is able to resolve about 3 poles (where *κ*_1_=10^3^, *κ*_2_=10^2^, *λ*_1_ = 10^12^, and *λ*_2_ = 10^6^) with the positions of the larger poles again beginning to degenerate first.

## 4. Summary

The idea of root projection (RP) was introduced to permit least-squares modification of a one-sided time series allowing a set of given complex numbers to be roots of the *Y* transform of the time series. The general framework of the method was presented, and techniques were given to adapt the method to the Gram-Schmidt algorithm. For time and frequency least-squares weighting, a dynamically dimensioned form of the Gram-Schmidt algorithm has been developed to carry out root projection. The code has been employed for root projection on time series with up to 1600 points and achieved better than 12 place accuracy. Illustrative examples of root projection were shown for noise reduction and filter construction as well as a least-squares extension of Prony’s method.

## Figures and Tables

**Figure 5a:**
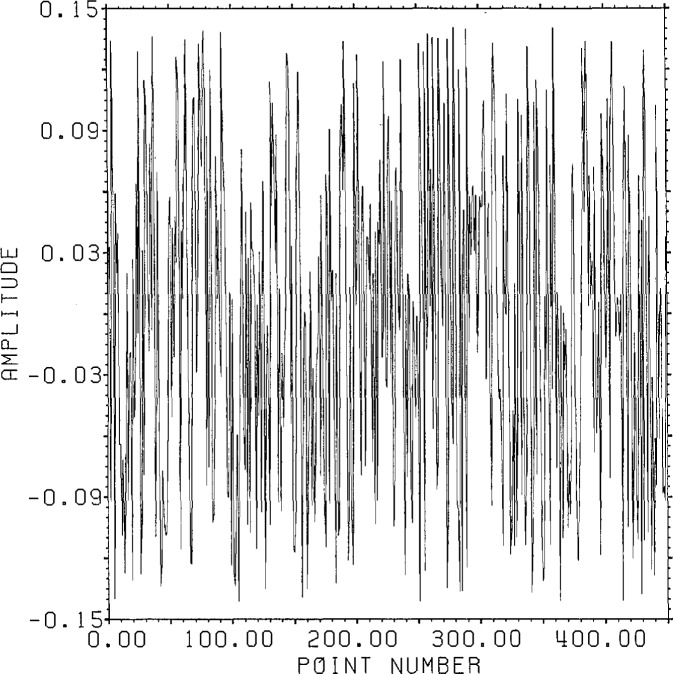
Noise added to series in figure 2.3a of reference [1].

**Figure 5b:**
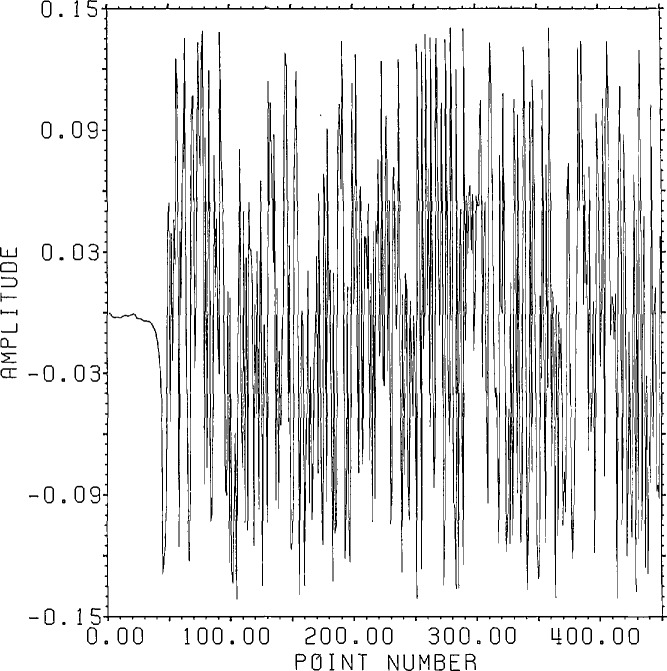
Noise after filtering using causal boundary roots of the series in figure 2.3a of reference [1] showing extensive front end noise reduction.

## Footnotes

1 A concise exposition of the linear algebra required for root projection, with *G =I* can also be given in terms of the QR decomposition [4] (although we use the “transposed” form of that in [4]).
